# Cytochrome P450 2C19 enzyme, Cytochrome P450 2C9 enzyme, and Cytochrome P450 2D6 enzyme allelic variants and its possible effect on drug metabolism: A retrospective study

**DOI:** 10.1097/MD.0000000000024545

**Published:** 2021-03-19

**Authors:** Domas Naujokaitis, Virginija Asmoniene, Edmundas Kadusevicius

**Affiliations:** a Faculty of Pharmacy, Lithuanian University of Health Sciences; b Department of Genetics and Molecular Medicine, Hospital of Lithuanian University of Health Sciences; c Institute of Physiology and Pharmacology, Faculty of Medicine, Medical Academy, Lithuanian University of Health Sciences, Kaunas, Lithuania.

**Keywords:** allelic variations, Cytochrome P450 2C19 enzyme, Cytochrome P450 2C9 enzyme, Cytochrome P450 2D6 enzyme, drug concentrations, drug metabolism

## Abstract

The objective of the present study was to assess the allelic variations of Cytochrome P450 (CYP) enzymes Cytochrome P450 2C19 (CYP2C19), Cytochrome P450 2C9 (CYP2C9), and Cytochrome P450 2D6 (CYP2D6) as they play a major role in drug metabolism. The interindividual genetic variabilities of these enzymes can account for different responsiveness as well as concentration fluctuations for a particular drug.

During the period of 2017 to 2018 a total of 54 patients have received pharmacogenetic testing at the Department of Genetics and Molecular Medicine at Kaunas Clinics. According to the genotype-metabolic phenotypes of CYP2C19, CYP2D6, CYP2C9 enzymes patients were classified according to the guidelines by Clinical Pharmacogenetics Implementation Consortium (CPIC): normal metabolizers (NMs), intermediate metabolizers (IMs), rapid metabolizers (RMs), ultrarapid metabolizers (UMs), and poor metabolizers (PMs).

CYP2C19 enzyme allelic distribution: 18 patients (33.33%) with ∗1/∗1 genotype were NMs; 14 patients (25.93%) with ∗1/∗2; ∗2/∗17 genotypes were classified as IMs; 15 patients (27.78%) possessed ∗1/∗17 genotype and were RMs; 4 patients (7.4%) had ∗17/∗17 genotype with increased enzyme activity compared with RMs, were classified as UMs; 3 patients (5.56%) had ∗2/∗2 genotype and were marked as PMs. CYP2D6 enzyme allelic distribution: 26 patients (48.148%) contained ∗1/∗1,∗2/∗2,∗1/∗2,∗1/∗41,∗2/∗41 genotypes with normal enzymatic function so were accounted as NMs; 21 patients (38.89%) with ∗1/∗5, ∗2/∗4, ∗10/∗41, ∗1/∗4, ∗1/∗3, ∗2/∗5, ∗2/∗4, ∗2/∗6 genotypes were accounted as IMs; 2 patients (3.7%) possessed ∗2XN genotype and were accounted as UMs and 5 patients (9.26%) possessed ∗4/∗5,∗4/∗10,∗4/∗9,∗4/∗41 genotypes and had non-functional enzymatic activity so were accounted as PMs; CYP2C9 enzyme allelic distribution: 44 patients (81.48%) with∗1/∗1 genotype were NMs; 10 patients (18.52%) with ∗1/∗2;∗1/∗3 genotypes were IMs.

The results of our study indicate that deviations from the normal enzymatic activity is common amongst Lithuanian people and combinatory genotyping of CYP2D6, CYP2C9, and CYP2C19 has to be promoted as an advanced method because of most commonly prescribed medicines like analgesics, antihypertensive, antidepressants are metabolized by multiple pathways involving enzymes in the *CYP450* family.

## 1 Introduction

The Cytochrome P450 (CYP450) enzyme superfamily plays a major role in the phase I metabolism of drugs.^[1]^ The enzymatic system can be divided into 18 families and 44 different subfamilies.^[2]^ In particular, enzymes Cytochrome P450 2C19 (CYP2C19), Cytochrome P450 2D6 (CYP2D6), and Cytochrome P450 2C9 (CYP2C9) are responsible for the metabolism of 35% to 45% of commonly prescribed drugs.^[3–7]^ For instance, CYP2C19 enzyme accounts for the metabolism of approximately 25 different drugs’ groups, while CYP2D6 enzyme takes part in drugs’ hydroxylation and demethylation of various clinically relevant drug groups.^[8]^ As for CYP2C9 enzyme it is important in the metabolism of 10% of clinically utilized drugs^[9]^ and is also named amongst one of the most clinically significant CYP isoenzyme.^[10]^

However, CYP2C19, CYP2D6, and CYP2C9 enzymes are encoded by genes where genetic polymorphisms are prevalent, thus causing altered or little to no enzyme activity.^[11]^ Such polymorphisms are influential in drug metabolism, the occurrence of adverse drug reactions and also to the efficacy of the treatment. A systematic review of 4139 studies has shown that adverse drug reactions may occur in 16.88% of patients during hospitalization period.^[12]^ Adverse drug reactions are responsible for 5% of all hospital admissions and almost 5% of patients experience adverse drug reaction while hospitalized. Moreover, adverse drug reactions cause 197,000 deaths annually in Europe.^[13]^ In addition, 70% of adverse drug reactions may be avoided through improved prescribing practices.^[14]^ The occurrence of such events indeed can be diminished by initiating further pharmacogenetic assays.^[15]^

Clinical pharmacogenetics implementation consortium (CPIC) guidelines^[16]^ aid clinicians in understanding how genetic test results should be utilized to maximize therapeutic efficacy. We do believe that following such guidelines when facing a resistant form of a particular disease should become mandatory, as well as entering into a particular regimen for patients with abnormal responsiveness to treatment with drugs shown in Table 1.

**Table 1:** The list of drug substrates which are partially or fully metabolized by CYP2C19, CYP2D6, and CYP2C9 enzymes.

CYP2C19 substrates	CYP2D6 substrates	CYP2C9 substrates
PPIs: Esomeprazole Lansoprazole Omeprazole Pantoprazole Anti-epileptics: Diazepam Phenytoin Phenobarbitone Carisoprodol Antidepressants: Amitriptyline Clomipramine Citalopram Imipramine Antifungals: Voriconazole Antineoplastic agents: Cyclophosphamide Antimalarial agents: Proguanil Beta blocking agents: Labetalol Platelet aggregation inhibitors: Clopidogrel	Antidepressants: Amitriptyline Citalopram Clomipramine Desipramine Doxepine Duloxetine Fluoxetine Fluvoxamine Imipramine Maprotiline Mianserin Notriptyline Paroxetine Venlafaxine Beta adrenergic blocking agents: Carvedilol Metoprolol Timolol Antipsychotics: Aripiprazole Chlorpromazine Clozapine Haloperidol Risperidone Perphenazine Tioridazine Zulopenthixol Antitussive agents: Dextrometorphan Antiemetics: Ondansetron Antiarrhytmic agents: Flecainide Metoprolol Antineoplastic agents: Tamoxifen Opioid analgesics: Codeine Tramadol Morphine	Angiotensin II blockers: Irbesartan Losartan Anticoagulants: Warfarin Anti-epileptic agents: Valproic acid Diuretics: Torsemide Leukotriene antagonists: Zafirlukast Statin: Fluvastatin NSAIDs: Celecoxib Diclofenac Ibuprofen Naproxen Piroxicam Hypoglycemic agents: Glimepiride Glipizide Tolbutamide Rosiglitazone Statins: Fluvastatin

CYP2C19 = Cytochrome P450 2C19 enzyme, CYP2C9 = Cytochrome P450 2C9 enzyme, CYP2D6 = Cytochrome P450 2D6 enzyme.

Unfortunately, the dosages of most prescribed drugs do not always correlate with the genetic background nor the metabolic profiles of individuals, which can result in therapeutic failure. Since drugs are mainly given according to the results of large scale clinical trials interindividual aspects of dose adjustments are not accounted correctly.^[17]^ We aimed to outline the distribution of allelic variants of individuals in Lithuania and to pave the way for future larger-scale studies that would help to foresee the individual therapeutic progress based on particular genotype that each individual possesses. Since it has become possible to predict the relationship between genetic background and drug response of an individual it is vital to state that genomic research can aid clinicians to constitute an improved method for personalized health care.^[17]^ The one-dose-fits-all strategy is held as standard, thus leading to abnormal exposure of different drugs. Illustration of such example is Warfarin, where the dosage is subjective to clinical and genetic factors of an individual.^[18]^ Overall, responsiveness to agents can vary from 10% to 90%.^[19]^ This is common for β_2_ agonists since up to 50% of patients do not benefit from the treatment,^[14]^ thus increasing the chances of therapeutic failures. Patel et al^[20]^ screened 93 hospitalization events of elderly patients from which 18% were due to preventable therapeutic failures.

Study conducted on 424 individuals from different Lithuanian ethnic groups disclosed that Lithuanian population is homogenic meaning that it is genetically unique compared with other populations by aspects of adaptation, diet, immunity, and diseases.^[21]^ Thus farther studies of Cytochrome P450 allelic variations are important in promoting a rational pharmacotherapy solutions as well as supplementing the genetic data of Lithuanian population. To our knowledge, such type of study regarding affiliations between pharmacogenetic testing, enzymatic activity, conversion to metabolic phenotype, and the rate of drug metabolism, was never conducted in Lithuania.

## 2 Methods and patients

### 2.1 Study population

This is a population-based retrospective study using data withdrawn from Kaunas Clinics database. All pharmacogenetic tests for 54 patients were done at the Department of Genetic and Molecular Medicine at Kaunas Clinics. Pharmacogenetic testing was conducted for 54 patients during the period of 2017 to 2018. This study was approved by the Lithuanian University of Health Sciences Department of Bioethics, no. of approval BEC FF-28 and by the Research and Studies Coordination Department of the hospital of Lithuanian University of Health Sciences.

### 2.2 Patient identification

Genotyping of CYP2C19, CYP2D6, and CYP2C9 liver enzymes were done during the period from January 1, 2017 to January 1, 2018. The pharmacogenetic assay was performed on a total of 54 patients done either voluntarily in an outpatient setting or following their doctor's order. We attributed patients to 5 particular groups based on their CYP2C19, CYP2D6, and CYP2C9 liver enzymes allele's distribution, enzymatic functionality, and possible type of metabolism they possessed. As stated by CPIC: NMs that have combinations of normal function and decreased function alleles; IMs with combinations of normal function, decreased function, and/or no function alleles; PMs containing combination of no function alleles and/or decreased function alleles; RMs with combinations of normal function and increased function alleles; and UMs that have 2 increased function alleles, or >2 normal function alleles.^[10,22–24]^ As there are other guidelines to follow in such cases (e.g., Dutch Pharmacogenetics Working Group) we made a choice based on the sheer amount of information taken into account while forming the guidelines. Since CPIC guidelines take pre-clinical studies, case reports, and clinical studies of moderate to good quality into consideration as Dutch Pharmacogenetics Working Group (DPWG) considers only clinical studies excluding the pre-clinical and case reports. In addition, the updates of CPIC guidelines are made whenever new evidence, that can make an impact on the pharmacotherapy, arises. On the contrary, DPWG makes updates every 4 years^[25]^ making DPWG guidelines less consistent when compared with CPIC guidelines. Also, there is limited availability for DPWG guidelines in English besides the guidelines that were presented in 2011.

### 2.3 Genetic information withdrawal

The pharmacogenetic assay was performed on blood samples which were withdrawn from patients either voluntarily in an outpatient setting or by doctor's order. The genetic analysis was done by using AutoGenomics INFINITI analyser (Carlsbad, California, United States of America) with assays specific for CYP2C19, CYP2D6, and CYP2C9 enzymes. Four milliliter of blood samples were taken to a BD Vacutainer, which contained potassium ethylenediaminetetra-acetic acid (K-EDTA) as an anticoagulant and were immediately frozen at –80 °C. Blood samples were thawed at room temperature and transferred in a 50 mL Falcon tube, which contained lysis buffer (0.32 M sucrose, 10 mM Tris–HCl pH 7.5, 5 mM MgCl_2_, 1% Triton X100), afterwards mixed gently and centrifuged. Two volumes of Fisio buffer (75 mM NaCl, 25 mM EDTA pH 8.0) were added to the pellet and centrifuged. After centrifugation, 1 volume of Fisio buffer (75 mM NaCl, 25 mM EDTA pH 8.0) was added and centrifuged. After the discharge of supernatant 0.6 volumes of buffer A (10 mM Tris–HCl pH 8.0, 50 mM K_2_EDTA pH 8.0), 40 μL of 10% Sodium dodecyl sulfate, and 12 μL proteinase K were added and incubated at 65 °C for 60 minutes. The concentration of deoxyribonucleic acid (DNA) was quantified by the absorbance measurement at 260 and 280 nm. At the same day, magnetic DNA extraction was made by using the manufacturer's protocol. Other steps were performed using the AutoGenomics INFINITI system. The hybridization of the amplified gene was done using the assay-specific Biofilm Chip Microarrays for different CYP enzymes. Biofilm Chip Microarray consists of polyester film coated with proprietary multi-layer components designed for DNA analysis, to determine the genetic polymorphisms in specific gene regions. Analysis within AutoGenomics INFINITI platform was done by using INFINITI PLUS Analyzer, which is responsible for integration, sample handling, detection, and results analysis. Intellipac Reagent Module was provided by the AutoGenomics INFINITI system, which acts as a communication link containing up to 4 reservoirs that house the test reagents and has an integrated memory chip. Further calculations concerning distribution of alleles were done by using Microsoft Excel 2013 version 15.0.4454.1503MSO 15.0.4481.1001.

## 3 Results

In the group of 54 patients that underwent the test, we observed all 5 functional groups of CYP2C19 liver enzymes. Eighteen patients (33.33%) which possessed CYP2C19 ∗1/∗1 genotype had fully functional enzymatic activity and thus were marked as NMs. Fourteen patients (25.93%) which possessed ∗1/∗2 and ∗2/∗17 genotypes had decreased enzyme activity thus were identified as intermediate metabolizers (IMs).^[26]^ Fifteen patients (27.78%) had a ∗1/∗17 genotype and altered enzyme activity when compared with normal metabolizers (NMs) indicating the marking of rapid metabolizers (RMs). Four patients (7.4%) had CYP2C19 ∗17∗/17 genotype which marked the alteration of enzymatic activity compared with RMs thus they were accounted as ultrarapid metabolizers (UMs). Three patients (5.56%) who possessed a genotype ∗2/∗2 had little to no enzymatic activity and were classified as poor metabolizers (PMs). The results of CYP2C19 enzyme genotyping are shown in Table 2.

**Table 2:** Detailed presentation of CYP2C19 genotype, enzyme activity, metabolism type, and the possible effect on drug concentrations.

Number of patients (%)	Genotypes	Enzyme activity	Metabolism type	Possible effect on drug concentration
n = 18 (33.33%)	∗1/∗1;	Fully functional;	Normal metabolizers	Drug concentration will be therapeutic.^[12]^
n = 14 (25.93%)	∗1/∗2; ∗2/∗17	Decreased enzyme activity;	Intermediate metabolizers	Drug concentration will exceed the therapeutic range.^[11]^
n = 15 (27.78%)	∗1/∗17	Altered compared to normal metabolizers;	Rapid metabolizers	Drug concentration may be subtherapeutic.
n = 4 (7.4%)	17/∗17	Altered compared to rapid metabolizers;	Ultrarapid metabolizers	Drug concentration will be subtherapeutic.^[12]^
n = 3 (5.56%)	∗2/∗2;	Little to no;	Poor metabolizers	Drug concentration will exceed the therapeutic range.^[9,10]^

CYP2C19 = Cytochrome P450 2C19 enzyme.

The assessment of the pharmacogenetics test results of CYP2D6 enzyme identified 4 functionality groups. Twenty six patients (48.15%) had ∗1/∗1, ∗2/∗2, ∗1/∗2, ∗1/∗41, ∗2/∗41 genotypes with functional enzyme activity and were accounted as NMs. Twenty one patients (38.89%) possessed ∗1/∗5, ∗2/∗4, ∗10/∗41, ∗1/∗4, ∗1/∗3, ∗2/∗5, ∗2/∗4, ∗2/∗6 genotypes with decreased enzyme activity and were marked as IMs. Two patients (3.7%) possessed ∗2xN genotype with altered enzymatic function compared with RMs thus were accounted as UMs. Five patients (9.26%) with genotypes ∗4/∗5, ∗4/∗10, ∗4/∗9, ∗4/∗41 had little to no enzymatic activity thus were marked as PMs. We did not find any patients which could be accounted as RMs. The detailed results of enzyme CYP2D6 allelic distribution, enzymatic activity, and possible impact on drugs metabolism are shown in Table 3.

**Table 3:** Detailed presentation of CYP2D6 genotype, enzyme activity, metabolism type, and the possible effect on drug concentrations.

Number of patients (%)	Genotypes	Enzyme activity	Metabolism type	Possible effect on drug concentration
n = 26 (48.15%)	∗1/∗1,∗2/∗2,∗1/∗2,∗1/∗41,∗2/∗41	Fully functional;	Normal metabolizers	Drug concentration will remain in the therapeutic range.
n = 21 (38.89%)	∗1/∗5,∗2/∗4,∗10/∗41,∗1/∗4,∗1/∗3,∗2/∗5,∗2/∗4,∗2/∗6;	Decreased;	Intermediate metabolizers	Drug concentration will exceed therapeutic range.
n = 2 (3.7%)	∗2XN;	Altered compared to rapid metabolizers;	Ultrarapid metabolizers	Drug concentration will be subtherapeutic.
n = 5 (9.26%)	∗4/∗5,∗4/∗10,∗4/∗9,∗4/∗41;	Little to no;	Poor Metabolizers	Drug concentration will exceed therapeutic range.

CYP2D6 = Cytochrome P450 2D6 enzyme.

The tests of CYP2C9 enzyme confirmed that patients had only normal and intermediate metabolic profile. Forty four patients (81.48%) had ∗1/∗1 genotype with fully functional enzyme activity and were marked as NMs. Ten patients (18.51%) possessed ∗1/∗2; ∗1/∗3 genotypes with decreased enzyme activity and were IMs. Allele distribution of CYP2C9 enzyme did not conclude any number of individuals that could be accounted for as RMs, Ums, and PMs. The results of CYP2C9 enzyme test results are presented in Table 4.

**Table 4:** Detailed presentation of CYP2C9 genotype, enzyme activity, metabolism type, and the possible effect on drug concentrations.

Number of patients (%)	Genotypes	Enzyme activity;	Metabolism type	Possible effect on drug concentration
n = 44 (81.48%)	∗1/∗1;	Fully functional;	Normal metabolizers	Drug concentration will remain in the therapeutic range.
n = 10 (18.51%)	∗1/∗2;∗1/∗3;	Decreased;	Intermediate metabolizers	Drug concentration will exceed therapeutic range.

CYP2C9 = Cytochrome P450 2C9 enzyme.

## 4 Discussion

Our study showed a significant number (35.18%) of individuals that were accounted as CYP2C19 UMs and RMs. Other larger scale multi-ethnic studies indicated that these particular variations of phenotypes are quite common. Mainly, these phenotypes are frequent in European populations since the results in different studies vary between 19% and 32%^[27–30]^ given the number of tested individuals and the region the study was taken in. Since CYP2C19 exhibits a high frequency of genetic polymorphisms that affects the drug exposure of many clinically significant drugs^[23]^ such as antidepressants, benzodiazepines, proton pump inhibitors (PPIs), and clopidogrel all of which are enlisted in Table 1. Wistfully, 25% of people taking clopidogrel experience a subtherapeutic effect because of genetic background and the imbalance of plasma concentration, ischemic events may reoccur.^[31]^ The interindividual dosage selection for clopidogrel can help prevent unwanted cardiac clinical symptoms.^[32]^ In ELEVATE-TIMI 56 trial individuals with 1 or 2 little to no function alleles could not generate pharmacologically active metabolite of clopidogrel and had worse therapeutic outcomes compared with normal function allele carriers.^[33]^ This indicates the importance of genotyping. Because of the ability to foresee the genetic background of an individual it is possible to establish a more potent way of prescribing practice and lower the risk of therapeutic failures.

Our study results confirmed, that NMs and IMs were the most common metabolic profiles of CYP2D6 enzyme and made up (87.04%) combined altogether. When compared with the worldwide distribution of CYP2D6 genotype, metabolic phenotype and enzymatic activity >80% of Europeans pose a normal allelic variant, 10% impaired, and about 3% contain an increased enzymatic activity containing alleles.^[34]^ Almost 5.5% of individuals in Western Europe countries contain the UM phenotype^[35]^ when compared with 3.7% in Lithuania. To express the significance of such enzyme it is beneficial to outline that CYP2D6 enzyme is responsible for 25% of clinically widely used drugs. Different diplotype variations can indeed affect the therapeutic outcome as well as the reoccurrence of adverse drug reactions of 50% of these drugs.^[36]^ It is worth noting that physicians should weigh the risks and benefits when including drugs that can be affected by existent polymorphism. For example, opioid analgesic Codeine can be held as a great example when genetic polymorphisms can influence occurrence of adverse drug reactions. The lack of analgesic effect of Codeine for PMs occurs in 7% of Caucasians and UMs are vulnerable to opioid toxicity, resulting in fatal events. Furthermore, a study done on CYP2D6 polymorphisms effect on risperidone treatment showed that PMs tend to have more serious adverse drug reactions.^[37]^ We believe that genotyping is advised when including agents (shown in Table 1) into therapeutic regimen. Furthermore, if the patient is given another agent which can interact within the alternative metabolic pathway, induce or inhibit the isoenzyme, significant drug–drug interactions may occur. In that way, it is important to understand that agents listed in Table 1 can have the ability to act on multiple enzymatic pathways, thus inducing or inhibiting the metabolism of other given drugs.

In regard to the CYP2C9 enzyme it is clearly seen that distribution size across the tested population is much smaller as compared with CYP2C19 or CYP2D6 enzymatic profiles. In detail 81.48% (44 patients) were NMs and 18.51% (10 patients) had intermediate metabolic profile IMs. As seen in other larger scale worldwide allelic distribution studies the population of Europeans tend to have only a small percentage of deviations from the normal enzymatic activity.^[29,38]^ If genotype distribution in the different European population is taken into perspective, ∗1/∗1 is the most frequent amongst Italian population with 65%. ∗1/2 and ∗1/∗3 genotypes make 29.3% combined in Italian population^[35]^ while our study presented (81.48%) and 18.51% of ∗1/∗1 and ∗1/∗2 and ∗1/∗3 diplotypes, accordingly. It is noteworthy that CYP2C9 along with vitamin K epoxide reductase complex subunit 1 (VKORC1) genotypes are the most notable genetic determinants for Warfarin, the widely used anticoagulant. Warfarin is notable for its’ narrow therapeutic index also it has the potential to cause severe adverse drug reactions leading to hospitalization.^[18]^ The genetic testing is worthy in patients who are about to undergo therapy with warfarin since it can prevent unexpected bleeding events.^[39]^ The European Pharmacogenetics of Anticoagulant Therapy (EU-PACT) presented that genotype-guided dosing of warfarin was beneficial when compared with standard clinical dosing in maintaining patients within an INR range of 2 to 3 over 3 months.^[37]^ The occurrence of unwanted events can be lowered by getting acquainted with drug-substrates of CYP2C9 enzyme as (shown in Table 1).

With this retrospective study, we wanted to emphasize the genotype–phenotype distribution amongst the Lithuanian population also display the effect that each metabolic abnormality can have on drug metabolism. Furthermore, to highlight the importance of personalized medicine in everyday healthcare and the positive effects it can possess, such as the prevention of therapeutic failures as well as limiting the occurrence of adverse drug reactions. We assent with van Puijenbroek et al,^[40]^ that adverse drug reaction can be held as a marker for pharmacogenetics testing. In addition, pharmacogenetic testing is useful when including agents with a narrow therapeutic index, where monitoring of adverse drug reactions is complex and where there is variability in drug response.^[41]^

Our study has several limitations. First, we think that a larger scale study in Lithuania can potentially disclose different variations in between genotype–phenotype conversion that can contribute to more positive therapeutic outcomes, the minimization of therapeutic failures as well as the increased utilization of personalized medicine. Nonetheless, the measurement of pharmacokinetic parameters by paying attention to individual genotype can help in improving the novel methods of personalized medicine.

Secondly, the cases where drugs metabolized by CYP2C19, CYP2D6, and CYP2C9 enzymes need to be accounted to be precise in foreseeing the uprise of positive therapeutic outcomes and the decline of adverse drug reactions with the help of pharmacogenetic testing.

## 5 Conclusions

Our study outlines that deviations from the normal enzymatic activity is common amongst Lithuanian population and genotyping of CYP2D6, CYP2C9, and CYP2C19 has to be promoted as an advanced method of personalized medicine because of most commonly prescribed medicines like analgesics, antihypertensive, antidepressants are metabolized by multiple pathways involving enzymes in the *CYP450* family. Drug metabolism indices for pharmacogenetics functional status, based on this, multigene model have to be developed and tested in clinical settings such as those involving pain, psychiatric disorders, and dyslipidaemias.^[42]^

Nonetheless, the pharmacogenetic testing is a powerful tool of personalized medicine which can affect patient and physician tremendously in prescribing right medicine with the right dose to the patient and achieving a positive therapeutic outcome.

## Author contributions

**Conceptualization:** Edmundas Kadusevicius.

**Data curation:** Virginija Asmoniene, Edmundas Kadusevicius.

**Formal analysis:** Virginija Asmoniene.

**Investigation:** Domas Naujokaitis, Virginija Asmoniene, Edmundas Kadusevicius.

**Methodology:** Domas Naujokaitis, Virginija Asmoniene, Edmundas Kadusevicius.

**Supervision:** Virginija Asmoniene, Edmundas Kadusevicius.

**Writing – original draft:** Domas Naujokaitis.

**Writing – review & editing:** Virginija Asmoniene, Edmundas Kadusevicius.
